# Type 2 Diabetes Mellitus and Vertebral Fracture Risk

**DOI:** 10.1007/s11914-020-00646-8

**Published:** 2021-01-12

**Authors:** Fjorda Koromani, Samuel Ghatan, Mandy van Hoek, M. Carola Zillikens, Edwin H. G. Oei, Fernando Rivadeneira, Ling Oei

**Affiliations:** 1 Department of Internal Medicine, Erasmus University Medical Center, PO Box 2040-Na27-24, 3000 CA Rotterdam, The Netherlands; 2 Department of Radiology and Nuclear Medicine, Erasmus University Medical Center, Rotterdam, The Netherlands; 3 Department of Epidemiology, Erasmus University Medical Center, Rotterdam, The Netherlands; 4 Department of Internal Medicine, Leiden University Medical Center, Leiden, The Netherlands

**Keywords:** Vertebral fractures, Diabetes, Osteoporosis, BMD, TBS

## Abstract

**Purpose of Review:**

The purpose of this review is to summarize the recently published evidence concerning vertebral fracture risk in individuals with diabetes mellitus.

**Recent Findings:**

Vertebral fracture risk is increased in individuals with T2DM. The presence of vertebral fractures in T2DM is associated with increased non-vertebral fracture risk and mortality. TBS could be helpful to estimate vertebral fracture risk in individuals with T2DM. An increased amount of bone marrow fat has been implicated in bone fragility in T2DM. Results from two recent studies show that both teriparatide and denosumab are effective in reducing vertebral fracture risk also in individuals with T2DM.

**Summary:**

Individuals with T2DM could benefit from systematic screening in the clinic for presence of vertebral fractures.

## Introduction

Osteoporosis and diabetes mellitus are diseases with a high prevalence and both substantially contribute to the disability burden worldwide [1, 2]. Diabetes mellitus is currently the fourth leading cause of disability worldwide, and in 2016, diabetes mellitus was the fifteenth leading cause of early death, forecasted to be seventh by 2040 [2]. Similarly, osteoporotic fractures are common, with an estimated nine million osteoporotic fractures in the year 2000 [3, 4]. Vertebral fractures, the most common type of osteoporotic fractures, are associated with an increased risk of mortality and non-vertebral fractures [5–7]. Despite the morbidity and mortality risk associated with occurrence of vertebral fractures, they remain underdiagnosed [8]. Type 1 diabetes mellitus (T1DM) and type 2 diabetes mellitus (T2DM), the two most common types of diabetes mellitus, have convincingly been shown to be associated with non-vertebral fracture risk in multiple large prospective studies and meta-analyses [9–13]. However, the evidence concerning *vertebral* fracture risk in individuals with diabetes mellitus, especially T2DM, has been less conclusive, providing results ranging from lower to no association, and to increased risk [14–19]. There are biological differences between vertebral fractures and non-vertebral fractures; these differences originate mainly from different bone material, biomechanical and functional properties of the trabecular and cortical bone. Vertebrae are composed predominantly of trabecular bone surrounded by a thin layer of cortical bone, in contrast to long bones where this layer is much thicker and the trabecular compartment is less prominent. Furthermore, trabecular bone is substantially more metabolically active and holds distinct biomechanical properties from cortical bone. Individuals with T1DM have decreased BMD, whereas individuals with T2DM have increased BMD [20]. Traditional diagnostic tools that incorporate BMD in fracture risk prediction tend to underestimate fracture risk in individuals with T2DM [21•]. Not only changes in bone mass but also changes in bone micro architecture can cause bone fragility.

The purpose of this review is to summarize the recently published evidence concerning vertebral fracture risk among individuals with diabetes mellitus. To decrease subjectivity and the chance of missing the inclusion of studies, we performed a sensitive systematic search of the last 5 years of literature in PubMed with search terms as follows: ((“Spinal Fractures”[Mesh]) AND (“Diabetes mellitus”[Mesh])) OR ((Diabet* [tiab]) AND (vertebr* [tiab]) AND (fract* [tiab])). The search resulted in 206 articles which were grouped into articles that analysed vertebral fracture risk in individuals with T1DM (*n* = 0), articles that analysed vertebral fracture risk in individuals with T2DM (*n* = 9), articles that concerned non-vertebral fracture risk and diabetes mellitus and possible pathophysiologic links between diabetes mellitus and fracture risk (*n* = 71) and studies that did not concern fracture risk in diabetes mellitus (*n* = 125). The literature was independently reviewed by FK and SG. The literature search showed no studies examining the risk of vertebral fractures in individuals with T1DM in the past 5 years; therefore, we re-did the search without the time constraint for T1DM. Interestingly, we could identify only two studies who had estimated vertebral fracture risk in individuals with T1DM. Therefore, this review is focused on recent findings concerning vertebral fracture risk in T2DM, and we have provided a short summary for risk of vertebral fractures in T1DM.

## Epidemiology of Vertebral Fractures in T1DM

We could identify only two older studies that have studied the risk of vertebral fractures among individuals with T1DM [10, 22]. A sex- and age-matched case-control study by Vestergaard et al. in a total of 498,617 Danish individuals from the general population found increased odds of vertebral fractures in individuals with T1DM compare to those without T1DM (OR = 2.48, 95% CI 1.33–4.62) [10]. Similarly, in a cross-sectional case-control study with significantly less participants (*n* = 164), Zhukouskaya et al. also reported increased odds of vertebral fractures among individuals with T1DM (OR = 4.20, 95% CI 1.40–12.70) [22].

## Epidemiology of Vertebral Fractures in T2DM

**Fig. 1:**
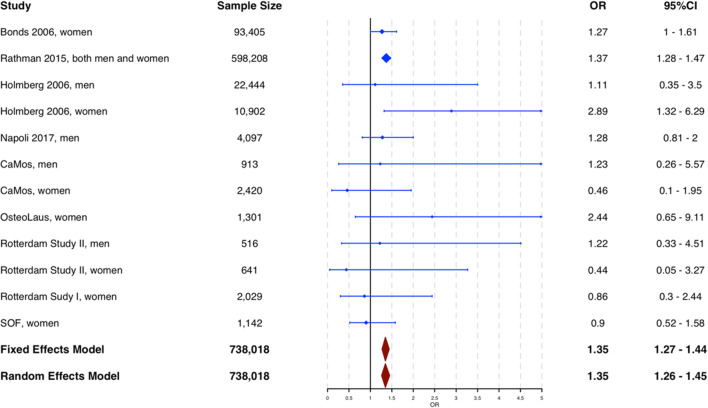
Forest plot of the meta-analysis across studies showing increased risk of incident vertebral fractures in individuals with T2DM. Figure adapted with permission [6]

The relationship between T2DM and vertebral fractures has been studied more extensively compared to T1DM; nevertheless, until recently the research had been inconclusive with meta-analyses characterized by high unexplained heterogeneity [14–19]. In our recent study, a meta-analysis of unpublished data from cohort studies and previously published studies, we performed two meta-analyses: one with both previously published and unpublished studies that had examined the relationship between T2DM and *prevalent* vertebral fractures (cross-sectional study design) and one with studies where the association between T2DM and *incident* vertebral fractures (either prospective or retrospective study design) was examined. In a sample of 37,292 individuals, those with T2DM had lower risk of *prevalent* vertebral fractures (OR 0.84 [95% CI 0.74–0.95]) without evidence of heterogeneity across studies. In a sample of 738,018 individuals, those with T2DM were at increased risk of *incident* vertebral fractures (OR 1.35 [95% CI 1.27–1.44]) with no evidence of heterogeneity across studies [6••] (Fig. 1). This difference in direction of effect seems to be explained by the observation that individuals with both T2DM and vertebral fractures have the highest mortality, and thus, prevalence studies for vertebral fractures in T2DM are impacted by survivorship selection bias.

## Risk Factors for Vertebral Fracture Risk in T2DM

Decreased BMD is strongly correlated with increased risk of vertebral fractures; however, in individuals with T2DM, BMD is on average higher than individuals without T2DM, suggesting that in T2DM, BMD underestimates the risk of fracture [20, 21•]. Trabecular bone score (TBS), a marker of deteriorated trabecular bone quality in patients with T2DM, has been postulated to be helpful in fracture risk assessment in patients with diabetes mellitus [23–25, 26•]. In our study, we reported that individuals with T2DM had higher BMD at the femoral neck and lumbar spine but lower TBS, compared to individuals without T2DM, independent of age, sex, medication use and BMI [6••]. Yamamoto et al. reported that prevalent vertebral fractures in individuals with T2DM were associated more strongly with TBS than BMD and suggested that an integrated assessment of bone strength by both BMD and TBS would help diagnose diabetic osteoporosis [27]. Choi et al. found indirect evidence that TBS and TBS-adjusted FRAX could be supplementary tools to predict osteoporotic fractures in T2DM, in a retrospective cross-sectional study of prevalent vertebral fractures in 169 Korean postmenopausal women [28]. Prediction studies on incident vertebral fractures are desirable. In the study by Zhukouskaya et al., TBS was found not to differ between individuals with or without T2DM and that a combination of TBS below 1.130 and BMD T-score below − 1.0 identified people with T2DM at high risk of vertebral fractures best [29]. These results need to be interpreted with caution, not only because the sample size is too small to draw solid conclusions but also because the thresholds used in the study are based on absolute values and will always be specific to the population studied and type of DXA used (as it is the case for TBS in this study) [29]. Similarly, results from the study by Chen et al. should be interpreted with caution, as they used cut-off values for TBS that are not derived from their study population. They reported that low bone mass and deteriorated TBS were noted in approximately two-thirds of women with T2DM and were also associated with vertebral fractures [30]. We could not find any published article estimating standardized clinical threshold values for TBS neither in individuals without T2DM nor in individuals with T2DM, and therefore, further research to define these thresholds is needed. BMI is a risk factor for vertebral fractures; however, the relationship is complex, with increased BMI correlated with increased bone mass but decreased bone quality [31]. Furthermore, studies estimating the association between BMI and vertebral fractures in individuals with T2DM are lacking. A study by Kanazawa et al. found that individuals with T2DM falling in the lowest and highest quartiles of BMI had increased risk of vertebral fractures compared to individuals in the middle quartile [32]. In our recent study, we found that T2DM was associated with increased vertebral fracture risk, only in the underweight category (BMI < 18.5 kg/m^2^) and with decreased risk of vertebral fractures in the obese category (BMI > 30 kg/m^2^) [6^••^]. Decreased muscle mass and mobility as well as increased frailty have been reported in individuals with T2DM compared to controls [33, 34]. Results from the CaMos cohort suggest that the increased frailty in individuals with T2DM could increase the risk of fragility fractures [34]. In the STRAMBO study, Schulz et al. found that in older men, moderate and severe vertebral fractures as well as T2DM were independently associated prospectively with lower grip strength, poor physical function, and higher risk of multiple falls [35]. In a small cross-sectional study among Japanese patients with T2DM, falling was correlated with vertebral fractures [36]. Vertebral fractures, even though they are often asymptomatic and go undetected, are strongly associated with increased incidence of any type of fracture [37]. In our recent study, we also examined whether the presence of vertebral fractures in individuals with T2DM predicts future non-vertebral fractures and whether the association differs by BMD levels [6••]. We found that compared to people without T2DM or vertebral fractures, those who had both T2DM and vertebral fractures had 2.5 times increased hazard ratio to acquire a non-vertebral fracture and a twofold increased hazard when compared to individuals with T2DM alone; the estimated increased risk did not vary when the analysis was stratified by clinical categories of BMD (i.e., normal, osteopenia and osteoporosis), suggesting that presence of vertebral fractures in individuals with T2DM captures an aspect of bone fragility different from that of BMD.

## Pathophysiology of Non-vertebral and Vertebral Fracture Risk in T2DM

A systematic review and meta-analysis conducted by Hygum et al. found that both bone formation and bone resorption markers are decreased in patients with T2DM, suggesting that low bone turnover might contribute to increased bone fragility in patients with T2DM [38]. In fact, growing evidence has accumulated to postulate that fracture risk in T2DM is a complication of inadequate glucose control [13, 39]. Several investigations have been performed to elucidate the molecular and biomechanical mechanisms underlying bone fragility in individuals with T2DM and have been described in detail elsewhere [40]. Levels of advanced glycation end products (AGEs) have been found to be increased in T2DM and might contribute to increased bone fragility through impaired enzymatic cross-linking and/or an increase in non-enzymatic cross-links in bone collagen [41]. In a recent study embedded in the Rotterdam Study, skin autofluorescence (SAF), a non-invasive biomarker of AGEs, was associated positively with prevalent vertebral fractures [42]. In cortical bone, compromised bone remodelling likely leads to accumulation of micro cracks, resulting in increased non-vertebral fracture risk [38–40]. While the processes affecting cortical bone are starting to be elucidated, evidence regarding the impact of low bone turnover on vertebral fracture risk is lacking. The thin cortices of vertebra and the integrity of the endplate have been shown to be critical for the assessment of vertebral fracture risk but much less is known about its underlying aetiology [43]. Most of the biomechanical support of vertebral bodies is believed to arise from the trabecular bone. This is in line with the finding that the TBS assessment does pick-up a biomechanical component not readily assessed by BMD. One of the potential factors that had been recently linked to decreased bone quality in individuals with T2DM is increased bone marrow fat. Free fatty acids released by adipocytes in bone marrow contribute in inhibiting osteoblast proliferation and function and inducing osteoblast apoptosis [44]. Elevated HOMA-IR was linked to higher marrow fat fraction in postmenopausal women with newly diagnosed T2DM independent of body composition [45]. Another study reported no association between bone marrow adipose tissue and bone density/strength nor with incident fractures. However, they reported that greater bone marrow fat was associated prospectively with greater loss of trabecular bone at the spine and femoral neck, and greater loss of spine compressive strength, in older women [46]. The study by Sheu et al. found significant inverse correlations between bone marrow fat estimated with MRI and BMD (− 0.30 for femoral neck and − 0.39 for total hip) among men with diabetes but not among those without diabetes [47]. None of the studies to date have examined whether bone marrow fat is associated with vertebral fracture risk.

## Vertebral Fractures in T2DM and Mortality

**Fig. 2:**
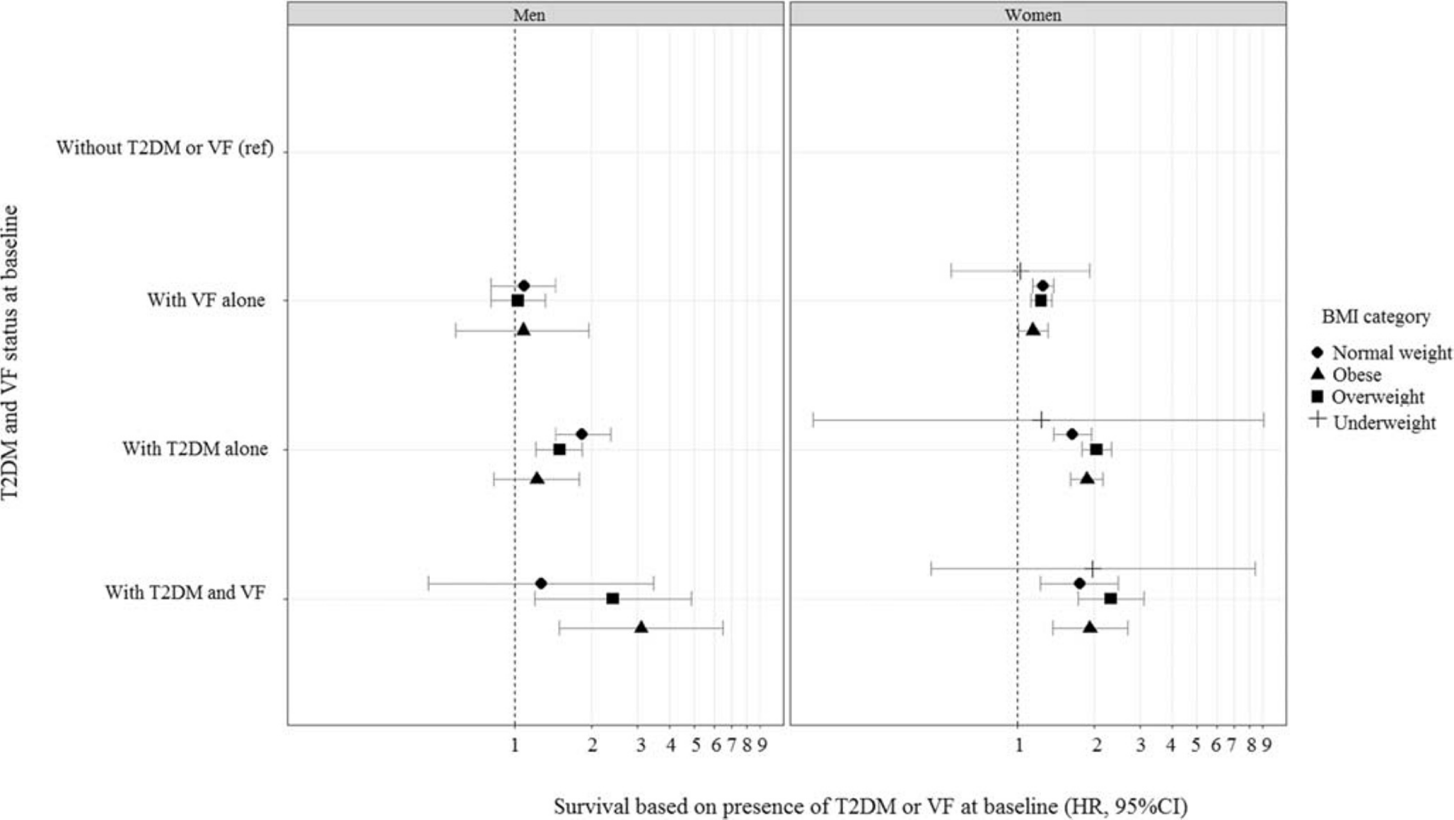
This graph shows mortality risk in men (*n* = 4405) and women (*n* = 15,417) with both vertebral fractures (VF) and type 2 diabetes mellitus (T2DM) stratified by BMI categories (underweight (< 18.5 kg/m^2^), normal weight (18.5–24.9 kg/m^2^), overweight (25.0–30.0 kg/m^2^) and obese (> 30.0 kg/m^2^)). The data points represent hazard ratios (HR) from Cox regression model adjusted for age (natural splines with 5 df), corticosteroid use, anti-osteoporotic treatment and cohort. The figure shows that obese men with T2DM with vertebral fractures have a strikingly high mortality hazard ratio compared to either individuals without T2DM or VF, with VF alone or with T2DM alone. Figure adapted with permission [6]

In the study by Koromani et al., sensitivity analyses showed that individuals with T2DM that were obese (BMI > 30.0 kg/m^2^) or were 74 years or older had the lowest prevalence of vertebral fractures [6••]. Subsequently, it was shown that individuals with both T2DM and vertebral fractures had increased mortality compared to individuals with vertebral fractures alone, or T2DM alone. Obese men with T2DM with vertebral fractures had a strikingly high mortality hazard ratio compared to obese men with vertebral fractures alone (HR = 3.10, 95% CI 1.25–7.57) and compared to obese men with T2DM alone (HR = 2.54, 95% CI 1.17–5.51) [6^••^] (Fig. 2). Similarly, in another study comprising 498,318 individuals whose information was extracted form GP databases in Spain, post-vertebral fracture mortality in individuals with T2DM was increased compared to individuals without T2DM (HR = 1.20, 95% CI 1.06–1.35) [48]. Also in a Japanese study of 411 T2DM patients, in those with grade 3 vertebral fractures, the mortality hazard rate was more than seven times the hazard rate of those without vertebral fractures (i.e. < grade 1) [49]. Presence of vertebral fractures in individuals with T2DM seems to be also a marker of increased frailty; yet more research is needed to identify causes of excess risk of mortality and on the effectiveness of measures to reduce post-fracture morbidity and mortality in individuals with T2DM.

## Anti-diabetes Medication and Vertebral Fracture Risk

The effect of anti-diabetes medication on any type of fracture risk is not established and shows a trend towards no statistically significant effect. In a nutshell, thiazolidinedione (TZD) and insulin have been implicated with increased fracture risk [50]. No significant effect on any type of fracture risk was found for metformin, sulfonylureas or DPP-4 inhibitors. Data concerning SGLT2 inhibitors are inconsistent showing increased or no effect on fracture risk, and currently there is insufficient data concerning GLP-1 receptor agonists’ effect on fracture risk [50]. Different types of anti-diabetes treatment may be associated with differences in risk of vertebral fractures (Fig. 1). In a nationwide study in Korea, 207,558 individuals on anti-diabetes medication were analysed to estimate whether the type of anti-diabetes treatment was associated with vertebral fracture risk. The authors reported that as compared to non-users, metformin + DPP4-inhibitor combination group had a trend towards a reduced vertebral fracture risk (HR = 0.73, *P* = 0.013) and showed a trend towards lower non-vertebral fracture risk compared to metformin + sulfonylurea group. From this perspective, they recommended clinicians to take these results of DPP4-inhibitors into consideration when prescribing diabetes medication, especially in elderly patients or those at high risk or fracture [51]. In contrast, another study reported that increased serum levels of DPP-4 in individuals with T2DM were associated with increased odds of vertebral fractures, but not with BMD [52]. Furthermore, Lin et al. reported that postmenopausal women with newly diagnosed T2DM have a higher marrow fat content compared to women without T2DM and that metformin treatment reduced marrow adiposity in T2DM [53]. A study in rats by Mai et al. suggests that metformin reduces RANKL, stimulates OPG expression in osteoblasts, inhibits osteoclast differentiation and prevents bone loss [54]. In a meta-analysis aiming to estimate the association between insulin use and risk of fracture, the authors found a trend towards increased risk of vertebral fractures among T2DM patients using insulin, compared to those using oral anti-diabetic treatment (OR 1.28, 95% CI, 0.90–1.81) [55]. Interestingly, in a study utilizing data from healthcare databases of the Italian region of Lombardy, the authors found that patients with T2DM who switched from oral medication to insulin were at increased risk of vertebral fractures (HR = 1.8, 95% 1.5–2.3) mainly in the period immediately after the start of insulin therapy. The observed association may result from higher hypoglycaemia; nonetheless, confounding by indication might play a role, because those individuals that have a necessity to switch to insulin might be those with more severe diabetes [56]. No vertebral fracture data are yet available for glucagon-like peptide-1 (GLP-1) receptor agonists, where bone turnover markers and BMD seem preserved in spite of weight loss. [57]

## Osteoporosis Medication and Vertebral Fracture Risk in Individuals with T2DM

Conversely, it is relevant to know if patients with diabetes-related bone disease benefit similarly of anti-osteoporotic medication as non-diabetic individuals with osteoporosis. Evidence in this area is still limited and largely focused on patients with T2DM. Bisphosphonates, the most widely used anti-osteoporotic therapy, have similar efficacy in individuals with or without diabetes. Also there is evidence suggesting that raloxifene has similar efficacy for prevention of vertebral fractures in both individuals with or without T2DM [58]. In a meta-analysis by Anagnostis et al., alendronate, raloxifene and teriparatide demonstrated comparable vertebral anti-fracture efficacy in individuals with or without diabetes [59]. Similarly, in the study by Langdahl et al., the authors aimed to assess fracture outcomes in subgroups of osteoporosis patients from four real-world teriparatide observational studies and found that similarly to individuals without diabetes, teriparatide also reduced clinical vertebral fracture risk in patients with T2DM [60]. A very recently published analysis of FREEDOM and FREEDOM extension found that denosumab in postmenopausal women with both osteoporosis and diabetes significantly increased BMD and decreased vertebral fracture risk [61].

A recent study using data from Medicare found that male sex, history of cerebrovascular accident/stroke and history of diabetes mellitus were risk factors associated with the lack of prescription of anti-osteoporosis medication within 1 year after a vertebral compression fracture. Moreover they reported a declining trend of anti-osteoporotic medication prescription during a 6-year interval (2008–2015) [62]. There are currently no guidelines on how to manage patients with T2DM who might be at risk for osteoporotic fractures. In a recent review, Ferrari et al. suggest to base the diagnosis of osteoporosis in individuals with T2DM on presence of fragility fracture and/or low BMD. They also recommend treatment thresholds to be applied similarly in patients with T2DM to those applied to the general population [63]. Furthermore, according to the most recent UK guidelines for prevention and management of osteoporosis, screening for vertebral fractures is recommended in patients with T2DM deemed as at high risk to acquire fractures such as postmenopausal women and older men with a height loss above 4 cm, presence of kyphosis or long-term use of glucocorticoids [64].

## Future Directions

In addition to the mechanisms described above, more data is needed on the complex pathophysiology that causes certain patients with T2DM to fracture and to be predisposed to increased mortality. Recently, the bone fragility in diabetes in Europe-towards a personalized medicine approach (FIDELIO) consortium has been founded. This research network will be a basis for application of next-generation techniques including investigations of the genetic epidemiological aspects and biological pathways of diabetic bone disease through Mendelian randomization. Other needs in the research field are head-to-head studies comparing the different anti-osteoporotic agents for their efficacy in preventing (vertebral) fractures in T2DM patients.

## Conclusions

Vertebral fracture risk is increased in individuals with T2DM. Furthermore, vertebral fractures in individuals with T2DM seem to be associated with increased mortality and non-vertebral fracture risk compared with individuals with T2DM and without vertebral fractures. Therefore, individuals with T2DM could benefit from systematic screening in the clinic for the presence of vertebral fractures. TBS seems to have utility in vertebral fracture risk assessment in individuals with T2DM; estimating standardized clinical threshold values for TBS could be of clinical value to improve fracture risk assessment in individuals with T2DM. Bone marrow fat assessment might also be useful for fracture risk assessment in individuals with T2DM; therefore, clinical or epidemiological studies are needed to estimate its utility. This might be difficult in clinical practice due to the need of MRI. Results from two recent studies show that both teriparatide and denosumab are effective in reducing vertebral fracture risk also in individuals with T2DM. Efforts to prevent skeletal complications and decrease their burden in patients with DM are needed.
